# Quantification and Further Refinement of Lateral Calcaneal Artery Flap for Complicated Heel Defect Reconstruction

**DOI:** 10.1097/GOX.0000000000004001

**Published:** 2022-02-28

**Authors:** Iftikhar Alam, Abdul Malik Mujahid

**Affiliations:** From the * Department of Burn and Plastic Surgery, Teaching Hospital/DG Khan Medical College, DG Khan, Punjab, Pakistan; † Department of Burn and Plastic Surgery, Teaching Hospital/DG Khan Medical College, DG Khan, Punjab, Pakistan.

## Abstract

**Background:**

Reconstruction of posterior heel defects requires tissues with adequate blood supply, good elasticity, and sensate; because of the traumatic origin in the young population, it ends up with morbidity. The lateral calcaneal artery is the arterial supply of the lateral calcaneal artery flap. This is one of the viable choices for coverage of hind foot soft-tissue defects. The objective of this study was to determine the outcome of the lateral calcaneal artery flap in terms of function, donor site morbidity, and duration of surgery.

**Method:**

This was a descriptive case series. Patients with complicated heel defects of small to medium size were included. The follow-up period was 6 months to 1 year of posttreatment. Outcome measures were noted and assessed in all patients in terms of function, donor area morbidity, and total duration of surgery. Significance was determined by assessing the gait, donor site pain, physical appearance at the surgical site, and time needed for surgery.

**Results:**

Twelve patients [men: eight (62.5%), women: four (37.5%)] were selected for this study, with their mean age being 14.4 ± 7.4 years. Mean defect size was (4.4 ± 0.5) cm. Mean flap size was 3.5–5.5 cm (*P* < 0.001). There was a near-normal gait in all patients, with a pain score of (0–2) on the visual analogue scale score, physical appearance by Vancouver scar scale score (0–3), and the mean duration of surgery was 45 ± 4.5 minutes.

**Conclusion:**

The lateral calcaneal artery flap is more effective for reconstruction of posterior heel and tendon Achilles defects in terms of function, donor area morbidity, and duration of surgery.

**Takeaways**

**Question:** Can the lateral calcaneal artery flap be used for reconstruction of small- to medium-sized complicated heel defects?

**Findings:** Outcome of lateral calcaneal artery flap for reconstruction of complicated heel defects in terms of function and donor site morbidity were assessed in a single-center case series.

**Meaning:** The lateral calcaneal artery flap could be the number one choice for small- to medium-sized heel defects.

## INTRODUCTION

Soft tissue defect of the posterior heel is the most challenging part of the leg because of the presence of bony prominence, specialized tissue characteristics, and the minimal availability of local tissue for coverage. This area is hard to reconstruct because of shearing forces and weight-bearing. The basic principle of plastic surgery is “replace like with like.”^1^ Options for soft tissue coverage can be skin graft, loco-regional flap, and free tissue transfer.^2^

There is always a search for new options to overcome these pitfalls. Grabb and Argent, in 1981, described the calcaneal artery flap, a viable option for soft tissue coverage of the posterior heel.^3^ The lateral calcaneal artery flap is an axial flap based on the perineal artery for coverage of defects of the Achilles tendon or plantar heel region. It may include the sural nerve for sensation. The flap has proved its reliability and effectiveness for coverage of tendo-achilli and posterior heel. Island,^4^ distally based,^5^ and free flaps are its modifications, all of which have a wide variety of clinical applications. Adipofascial modification was reported by Lin et al.^6^ They have used it for soft-tissue defects of the malleoli and tendo-achilli region. It has nonbulky, sensate cover and less donor site morbidity. The drawbacks of this flap are donor site contour deformity and hypothesis at the lateral portion of the foot. However, these complications gradually disappear.

Freeman et al^7^ observed that the lateral calcaneal artery (LCA) arises from the peroneal artery (80%) and from the posterior tibial artery (10%). The Achilles tendon location has been used as an anatomical landmark of the LCA by Grabb and Argent, but the variable thickness, blending to the calcaneal bone.^8^ In addition, the arc of the flap varies depending on the size of defect to be reconstructed.

The rationale of our study was that we can use this flap as the first choice for small- to medium-sized defects of the posterior heel, Achilles tendon, and lateral malleolus. The advantages of this flap are that it preserves the major artery of the foot, needs minimum operative time, and is relatively thin with less donor-site morbidity. Only limited local international data are available for this study. Successful outcome of our study will help us further endorse this flap to be used for small- to medium-sized defects of the posterior heel.

## PATIENTS AND METHOD

We carried out this study at the Burn and Plastic Surgery Department, Teaching Hospital DG Khan, from September 2019 to March 2021. Twelve patients (8 men‚ 4 women) with soft-tissue defects over tendo-achilli and posterior heel wound fulfilling inclusion criteria (soft-tissue defects caused by acute trauma complicated by infection and/or failed primary reconstruction and chronic nonhealing wound) were included in this study. Patients with peripheral vascular disease, those with a defect size greater than 7 cm, and patients with coagulopathy were excluded from our study. After obtaining approval from the hospital ethical committee, we received informed consent from all patients.

We took preoperative photographs to compare postoperative outcomes after baseline investigation and fitness for anesthesia, and operated on all the patients under general anesthesia. Debridement/wound excision was done, and the flap was marked according to defect dimensions. The size of flaps ranged from 3.5 × 2.0 cm to 5.5 × 3.5 cm, and patient ages ranged from 10 to 21 years (mean = 14 years). The follow-up period ranged from 6 months to 1 year. The chief surgeon (IA) did dissection up to the level of the lateral malleolus. Flap insetting was done with Polypropylene 4/0 suture. A thin split-thickness skin graft was taken from the lateral calf or thigh for donor site coverage and was immobilized with a bolster dressing. The patients were hospitalized, with their foot elevated for 3 days after surgery. Flap monitoring was done clinically for 5 days. The first dressing of the donor flap area was changed on the fifth day and after every third day up to 14 days‚ and then each month for 12 months. Patients were encouraged to walk after 3 weeks of wound healing. The consultant plastic surgeon (with 5-year postfellowship experience) noted and assessed outcome measures.

## RESULTS

**Fig. 1.**
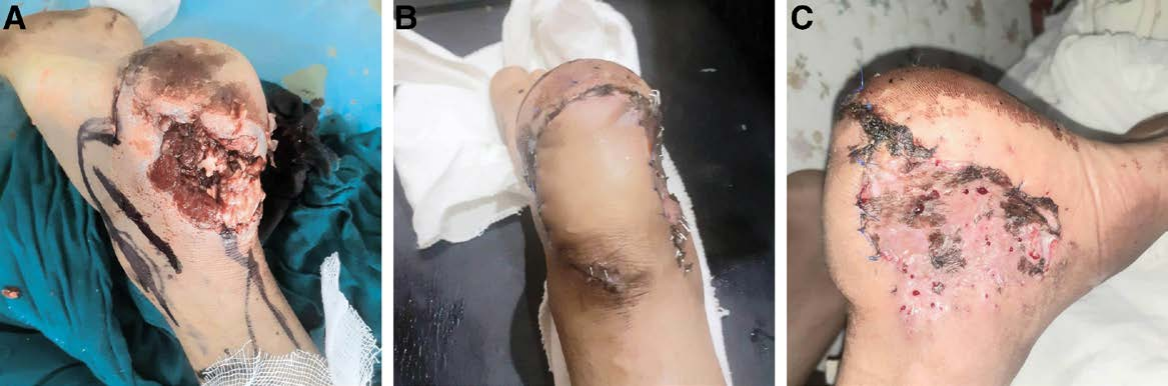
A, A complicated wound with infection, failed previous reconstruction, and Achilles tendon injury in a 12-year-old boy, after wound debridement exposure of the Achilles tendon. B, Postoperative results showing complete flap survival. C, Graft integrated on the donor site of flap.

**Fig. 2.**
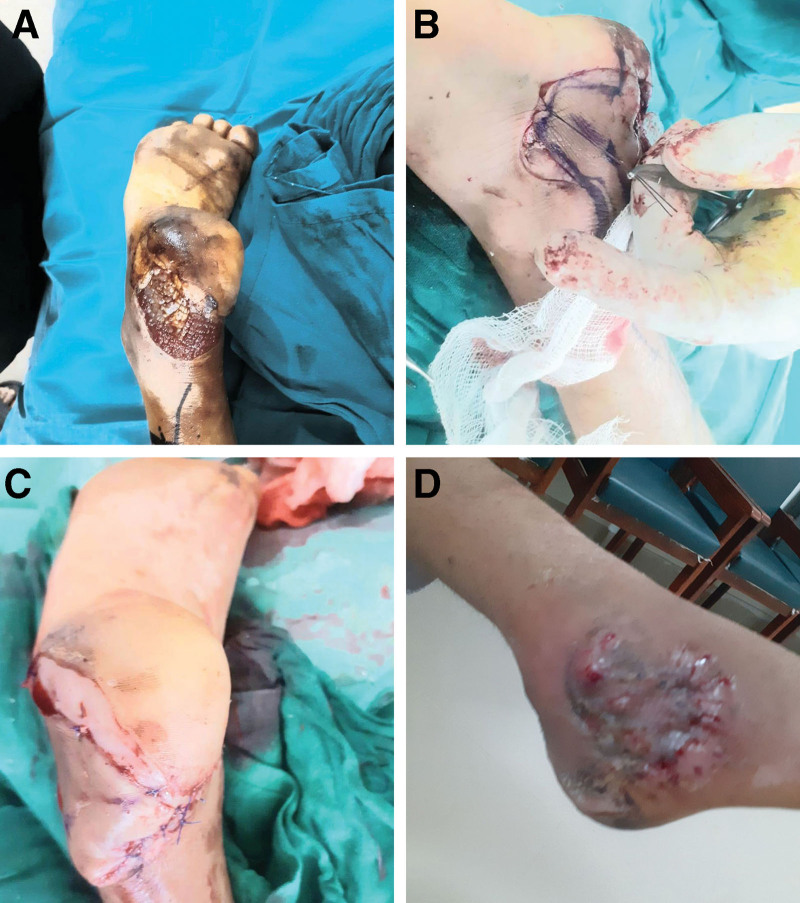
A, A complicated wound with infection, failed previous reconstruction, and tendo-achilli injury in an 18-year-old man, after wound debridement exposure of the Achilles tendon. B, Harvesting of flap. C, Postoperative results showing complete flap survival. D, Graft integrated on the donor site.

**Fig 3.**
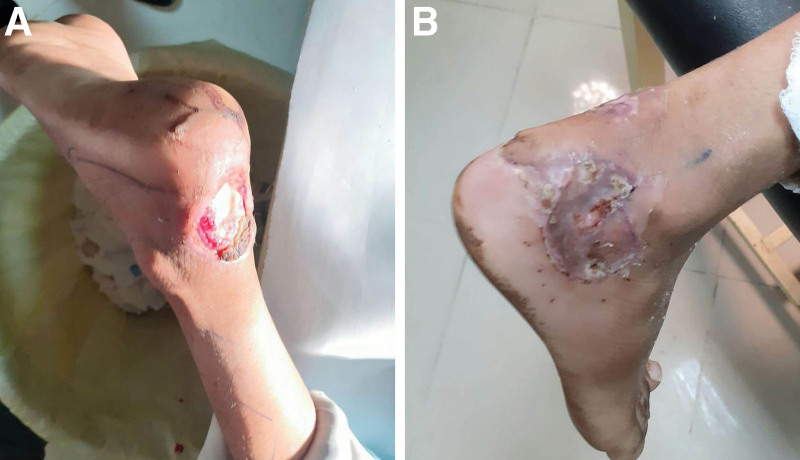
A, A traumatic wound with infection in a 10-year-old boy, after wound debridement exposure of the Achilles tendon. B, Postoperative results showing complete flap survival and graft take on the donor site.

**Fig. 4.**
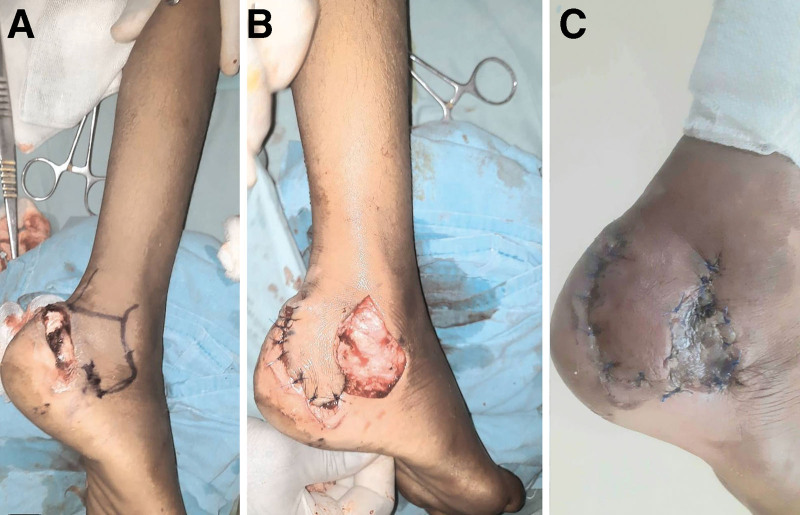
A, Nonhealing wound on posterolateral heel with exposed Achilles tendon and calcaneum wound after debridement. B, Flap marking. C, Flap fully survived and graft taken on donor area.

Table 1 shows the patient demographic data. All 12 flaps had good perfusion and survived fully; venous congestion was not observed, but flap swelling persisted for 3–5 days. The split-thickness skin grafts on the recipient site had taken well in all patients. There was a partial loss of the split-thickness skin graft in a single case, which healed in time with no need for secondary procedure, but developed hyperkeratosis at the junction of skin graft and planter skin, which was resolved by conservative treatment (Figs. 1–4). All patients were mobile after 3 weeks of surgery, and their foot motion was normal. There was no breakdown of the flap or the skin graft with regular footwear. Total operative time was 45 minutes to 1 hour (Table 1).

**Table 1.** Demographic and Data Interpretation

Case	Age of Patient	Etiology of Wound	Site of Defect	Flap Size	Follow-up Duration	Donor Area Complication	Functional Outcome (Gait with Shoe Wear)	Duration of Surgery	Vancouver Scar Scale, Donor Area
1	15	Trauma (complicated by infection)	Posterolateral heel	3.3 × 4 cm	1 y	No	Good	60 min	2
2	18	Trauma (failed repair and superadded infection)	Posterolateral heel	3.5 × 5 cm	1 y	No	Good	45 min	2
3	19	Trauma (superadded infection and failed repair)	Tendo-achilli	3.3 × 3.5 cm	1 y	No	Good	46 min	2
4	35	Trauma	Posterolateral heel	3.2 × 4.5 cm	1 y	No	Good	55 min	2
5	45	Nonhealing wound	Posterolateral heel	3.1 × 5.3 cm	1 y	Skin hyperkeratosis	Good	60 min	3
6	11	Trauma	Posterolateral heel	3.4 × 5.2 cm	1 y	No	Good	45 min	3
7.	19	Trauma (superadded infection and failed primary reconstruction)	Posterolateral heel	3.5 × 5 Cm	1 y	No	Good	55 min	3
8	35	Trauma (failed primary reconstruction)	Posterolateral heel	3.2 × 4.5 cm	1 y	No	Good	45 min	3
9	35	Trauma (superadded infection and failed repair)	Tendo-achilli	3.5 × 5.5 cm	9 mo	No	Good	55 min	2
10	35	Trauma (failed primary reconstruction)	Tendo-achilli	3 × 3.9 cm	9 mo	No	Good	45 min	3
11	11	Post-traumatic wound and failed primary reconstruction	Posterolateral heel	3.5 × 5 cm	9 mo	No	Good	50 min	3
12	45	Nonhealing wound	Posterolateral heel	3.7 × 5 cm	1 y	No	Good	60 min	3

## DISCUSSION

Posterolateral heel and Achilles tendon site defects often pose difficulty in their reconstruction because of high functional demands, poor vascularity, tendinous/bony bed, and frequent mobility. Conservative treatment is usually inadequate. Use of split-thickness or full-thickness skin grafts frequently leads to functionally and aesthetically unacceptable results, with off and on scar contracture/hypertrophy, while free flap transfers are technically challenging, requiring microsurgical expertise and a long duration of surgery, with chances of significant perioperative morbidity.^9^

The goal of reconstruction is to have supple soft-tissue coverage to help in skeletal reconstruction and to replace “like with like” when possible. It is necessary to preserve both motor and sensory functions to ambulate. To make the best reconstructive decision, tissue deficit (types of tissues lost), location, the defect size, length of vascular pedicle needed, and state of the wound should be considered. The foot is a difficult area to reconstruct and is prone to injury and disease. It is best to think about foot reconstruction by subunits to get the best functional and aesthetic results. Reconstruction of posterior heel defects requires tissues with adequate blood supply, good elasticity, and sensate; because of traumatic origin in the young population, it ends up with morbidity.

The lateral calcaneal artery is the arterial supply of the lateral calcaneal artery. This is one of the viable choices for hind foot soft tissue flap cover. Advantages of this flap include a high success rate, minimum postoperative morbidity, adequate soft tissue coverage, and good functional outcome.^10^ Investigation of lateral calcaneal flap morphology has led to its various modifications, which include island, adipofascial, reverse, and V-Y advancement types.

Anatomy of the flap has been studied in detail, and location of the vessel has been described, which are very helpful in planning the flap. Status of the lateral calcaneal artery must also be evaluated preoperatively by Doppler for safety. In one study, the lateral calcaneal artery flap proved to be effective for small-sized wounds in the plantar aspect of the heel with durable soft tissue cover.^11^ Free flaps are essential for wounds that are not amenable to loco-regional flaps for larger defects with poor vascularity and failed previous surgery. The sural artery perforator flap is ideal for patients with clinically free distal leg collateral blood supply and is a potential coverage option that does not require microsurgery expertise. Adipofascial form^12^ was reported by Lin et al, with intact donor site skin.

In our case series, we determined patient satisfaction based on the durability of the reconstruction, and the patient’s ability to use suitable footwear with or without the use of adjunctive appliances, walking activities, donor site morbidity, and the overall aesthetic outcome. The use of similar donor tissue to match the tissue of the recipient site was achieved by means of pedicle and island lateral calcaneal flaps, which proved to be very durable as a longstanding reconstruction. Durability of the reversed sural and free flaps was also apparent in regard to their supple tissue coverage. Although most of the patients who received the sural and free flaps had to wear larger-sized or custom-made shoes on the affected foot, they were, in all cases, able to ambulate without inhibition and without the need for crutches after the flaps had healed. During the follow-up period, none of these patients displayed in-situ infection or recurrent ulceration in the reconstructed tissues.

The silicone padding was used because it has been shown to decrease the vertical peak plantar pressure and shear forces while walking. Custom footwear, moreover, was employed in these patients because it has been shown to accommodate the reconstructed tissue, while decreasing pressure and shear forces applied to the skin overlying bone. Reconstruction of soft tissue defects at tendon Achilles and heel may pose a problem, as pressure on pedicle in supine position jeopardizes the blood supply. In such cases, the patient may simply be asked to rest in prone position and to avoid pressure on the pedicle. However, we took advantage of the positioning of the foot in various cases. As it is apparent, the cases requiring coverage with Achilles tendon repair may need plantar flexion for relieving tension on the pedicle. This measure was adopted with good results. A pillow beneath the foot with the patient in prone position suffices to hold the foot in plantar flexion. In any instance, direct pressure on the flap and its base was avoided.

Lateral calcaneal artery fasciocutaneous flaps should be considered in the armamentarium for complicated posterolateral heel defects.^13–15^ There is no loss of a major artery of the foot. This flap is relatively thin, with acceptable morbidity at the donor sites.

## CONCLUSIONS

The lateral calcaneal artery flap is more useful for coverage of posterior heel and tendon calcaneus defects in terms of function, donor area morbidity, and duration of surgery. It can be the number one choice for small- to medium-sized defects of the heel and Achilles.

## References

[1] Gillies H Millard DR. The Principles and Art of Plastic Surgery. Butterworth; 1957:48–54.

[2] Anwer A Anwar M Tariq M. Versatility of lateral calcaneal artery skin flap for coverage of posterior heel defects—our experience. Pak J Med Health Sci. 2013;7:679–683.

[3] Grabb WC Argenta LC. The lateral calcaneal artery skin flap (the lateral calcaneal artery, lesser saphenous vein, and sural nerve skin flap). Plast Reconstr Surg. 1981;68:723–730. 7291343 10.1097/00006534-198111000-00010

[4] Jyothsna B. Lateral calcaneal artery island flap. IOSR Journal of Dental and Medical Sciences (IOSR-JDMS). 2020;19:01–04.

[5] Li L Xue C Li J Zhang J. Application of flap in repair of heel skin and soft tissue. 2008;22:800–802. 18681278

[6] Lin SD Lai CS Chiu YT. The lateral calcaneal artery adipofascial flap. Br J Plast Surg. 1996;49:52–57. 8705103 10.1016/s0007-1226(96)90187-3

[7] Freeman B J Duff S Allen S. The extended lateral approach to the hind foot: anatomical basis and surgical implications. J Bone Joint Surg Br. 1998;80:139–143. 9460971 10.1302/0301-620x.80b1.7987

[8] Mackenzie DJ Seyfer AE. Reconstructive surgery: lower extremity coverage. In: Mathes SJ Hentz VR. Plastic Surgery. 2nd ed. Philadelphia, Pa.: Saunders Elsevier; 2006:1355–1381.

[9] El-Shazly M Yassin O Kamal A. Soft tissue defects of the heel: a surgical reconstruction algorithm based on a retrospective cohort study. J Foot Ankle Surg. 2008;47:145–152. 18312922 10.1053/j.jfas.2007.12.010

[10] Chauhan VS Gupta S. Lateral calcaneal artery flap: a versatile flap for coverage of posterior heel defects in moribund patients. Reconstr Surg Anaplastol. 2018;7:2161–1173.

[11] Chowdhury MA Sultana T Hossain S Islam S. Use of lateral calcaneal flap for the reconstruction of posterior heel defect. J Bangladesh Coll Phys Surg. 2020;38:116–120.

[12] Chung MS Baek GH Gong HS. Lateral calcaneal artery adipofascial flap for reconstruction of the posterior heel of the foot. Clin Orthop Surg. 2009;1:1–5. 19884990 10.4055/cios.2009.1.1.1 PMC2766687

[13] Wang SJ Kim YD Huang HH. Lateral calcaneal artery perforator-based skin flaps for coverage of lower-posterior heel defects. J Plast Reconstr Aesthet Surg. 2015;68:571–579. 25752717 10.1016/j.bjps.2014.12.027

[14] Elsaidy MA El-Shafey K. The lateral calcaneal artery: anatomic basis for planning safe surgical approaches. Clin Anat. 2009;22:834–839. 19637301 10.1002/ca.20840

[15] Islam MT Abdullah SN Uddin MJ Rahman TR. Use of lateral calcaneal flap for coverage of posterior heel defects. Mediscope. 2017;4:5–10.

